# Das Verbundprojekt „Personalisierte Medizin für die Onkologie“ (PM4Onco) als Teil der Medizininformatik-Initiative (MII)

**DOI:** 10.1007/s00103-024-03886-6

**Published:** 2024-05-13

**Authors:** Patrick Metzger, Melanie Boerries

**Affiliations:** 1 Institut für Medizinische Bioinformatik und Systemmedizin (IBSM), Universitätsklinikum Freiburg, Medizinische Fakultät, Universität Freiburg, Breisacher Straße 153, 79110 Freiburg, Deutschland; 2 Deutsches Konsortium für Translationale Krebsforschung (DKTK), Standort Freiburg, Kooperation zwischen DKFZ und Universitätsklinikum Freiburg, Universität Freiburg, Freiburg, Deutschland; 3 Comprehensive Cancer Center Freiburg (CCCF), Universitätsklinikum Freiburg, Universität Freiburg, Freiburg, Deutschland

**Keywords:** Präzisionsmedizin, Hochdurchsatzdaten, Datenstandards, Interdisziplinarität, Entscheidungsunterstützende Analysen, Precision medicine, High-throughput data, Data standards, Interdisciplinarity, Decision-supporting analyses

## Abstract

Das Verbundprojekt Personalisierte Medizin für die Onkologie (PM4Onco) wurde 2023 im Rahmen der Nationalen Dekade gegen Krebs (NDK) ins Leben gerufen und wird als Anwendungsfall der Medizininformatik-Initiative (MII) geführt. Es verfolgt das Ziel, eine nachhaltige Infrastruktur zur Integration und Nutzung von Daten aus der klinischen und biomedizinischen Forschung zu etablieren, und bündelt dabei die Erfahrungen und Vorarbeiten aller 4 Konsortien der MII sowie der onkologischen Spitzenzentren in Deutschland. Die von PM4Onco bereitgestellten Daten werden in geeigneter Form aufbereitet, um die Entscheidungsfindung in molekularen Tumorboards zu unterstützen. Dieses Konzept und die entsprechende Infrastruktur werden auf die 23 beteiligten Standorte ausgeweitet und damit wird ein verbesserter Zugang zu zielgerichteten Therapien ermöglicht, die auf klinischen Informationen und der Analyse molekulargenetischer Veränderungen in Tumoren in verschiedenen Krankheitsstadien basieren. Dies trägt dazu bei, die Behandlung und Prognose von Tumorerkrankungen zu verbessern.

Klinische Krebsregister sind Teil des Projekts, um die Datenqualität durch standardisierte Dokumentationsroutinen zu erhöhen. Klinische Expert:innen beraten bei der Erweiterung der Kerndatensätze für die Personalisierte Medizin (PM). Einen entscheidenden Beitrag liefern Informationen zur Lebensqualität und zum Behandlungserfolg, die Patient:innen über Fragebögen rückmelden und die bisher außerhalb klinischer Studien kaum erfasst werden. Patientenvertreter:innen begleiten das Projekt, um die wichtige Perspektive der Betroffenen in den Entscheidungen zu berücksichtigen. PM4Onco schafft somit eine Allianz zwischen MII, onkologischen Spitzenzentren, klinischen Krebsregistern, Nachwuchswissenschaftler:innen, Patient:innen und Bürger:innen, um die PM in der Krebstherapie zu stärken und voranzubringen.

## Abstract (en)

The collaborative project Personalized Medicine for Oncology (PM4Onco) was launched in 2023 as part of the National Decade against Cancer (NKD) and is executed within the Medical Informatics Initiative (MII). Its aim is to establish a sustainable infrastructure for the integration and use of data from clinical and biomedical research and therefore combines the experience and preliminary work of all four consortia of the MII and the leading oncology centers in Germany. The data provided by PM4Onco will be prepared in a suitable form to support decision making in molecular tumor boards. This concept and infrastructure will be extended to 23 participating partner sites and thus improve access to targeted therapies based on clinical information and analysis of molecular genetic alterations in tumors at different stages of the disease. This will help to improve the treatment and prognosis of tumor diseases.

Clinical cancer registries are involved in the project to improve data quality through standardized documentation routines. Clinical experts advise on the expansion of the core datasets for personalized medicine (PM). Information on quality of life and treatment outcomes reported by patients in questionnaires, which is rarely collected outside of clinical trials, will make a significant contribution. Patient representatives are involved from the onset to ensure that the important perspective of patients is taken into account in the decision-making process. PM4Onco thus creates an alliance between the MII, oncological centers of excellence, clinical cancer registries, young scientists, patients, and citizens to strengthen and advance PM in cancer therapy.

## Hintergrund

Die Personalisierte Medizin (PM) in der Onkologie hat in den letzten Jahren große Fortschritte gemacht und spielt eine entscheidende Rolle bei der individualisierten Behandlung von Krebserkrankungen. Ein Kernelement dieser Entwicklung sind die Molekularen Tumorboards (MTBs), die als interdisziplinäre Gremien die Grundlage für personalisierte Therapieentscheidungen bilden. Ziel der MTBs ist es, im Rahmen der Präzisionsonkologie potenziell wirksame Behandlungen für Patient:innen ohne herkömmliche oder erfolgversprechende Therapieoptionen bzw. mit seltenen Tumorerkrankungen zu identifizieren und diesen Patient:innen eine zielgerichtete Therapie im Rahmen klinischer Studien oder individueller Heilversuche anzubieten. Hierfür ist es notwendig, verschiedenste Datensätze wie klinische Daten und auch komplexe Omics-Informationen wie Genom- und Transkriptomdaten zu integrieren, bereitzustellen und visuell aufzubereiten. Dieser Aufgabe widmet sich das im Jahr 2023 im Rahmen der Nationalen Dekade gegen Krebs gegründete und als Anwendungsfall der deutschen Medizininformatik-Initiative (MII) geführte Verbundprojekt „Personalisierte Medizin für die Onkologie“ (PM4Onco).

In diesem Artikel werden zunächst Aufbau, Aufgaben und Ziele von Molekularen Tumorboards erklärt. Im Anschluss wird genauer auf das Verbundprojekt PM4Onco und seine Rolle bei der Bewältigung von Herausforderungen in der Personalisierten Medizin eingegangen. Die einzelnen Aufgabenbereiche in Form von „Arbeitspaketen“ werden beschrieben.

## Molekulare Tumorboards

Krebserkrankungen sind äußerst heterogen und lassen sich auf genetische Veränderungen in den Zellen zurückführen. Die Entschlüsselung des individuellen genetischen Profils eines Tumors ermöglicht es, gezielt auf spezifische molekulare Veränderungen zu reagieren [1–3] Hier setzt die Personalisierte Medizin (PM) an, deren Ziel es ist, auf Basis der genetischen Signaturen von Tumoren individuelle Therapieansätze zu entwickeln.

Eine entscheidende Rolle bei der Umsetzung der PM spielen Molekulare Tumorboards (MTBs). Die ersten MTBs wurden bereits etwa Mitte der 2010er-Jahre an einigen Unikliniken, wie z. B. in Heidelberg, Freiburg oder Tübingen, als Teil der Comprehensive Cancer Centers (CCCs) gegründet. Inzwischen sind MTBs an allen CCCs (aktuell 15 CCCs mit 25 Standorten) eingerichtet und etabliert, unterstützt durch Initiativen wie PM4Onco und z. B. das Deutsche Netzwerk Personalisierte Medizin (DNPM). Die MTBs setzen sich jeweils aus einem interdisziplinären Team von Spezialist:innen verschiedenster Fachrichtungen zusammen, das medizinische und naturwissenschaftliche Expertise mit translationaler Onkologie, Molekularbiologie und Bioinformatik verbindet [4–6]. In MTBs sind nicht nur internistische Onkologen vertreten, sondern auch Expert:innen aus verschiedenen klinischen Disziplinen wie Gynäkologie, Urologie, Gastroenterologie und Pulmologie. Hinzu kommen Vertreter:innen aus der Pathologie, Bioinformatik und Systemmedizin sowie Humangenetik, Radiologie, Nuklearmedizin und Expert:innen aus naturwissenschaftlichen Disziplinen wie Molekularmedizin und Biologie [6–9].

**Abb. 1:**
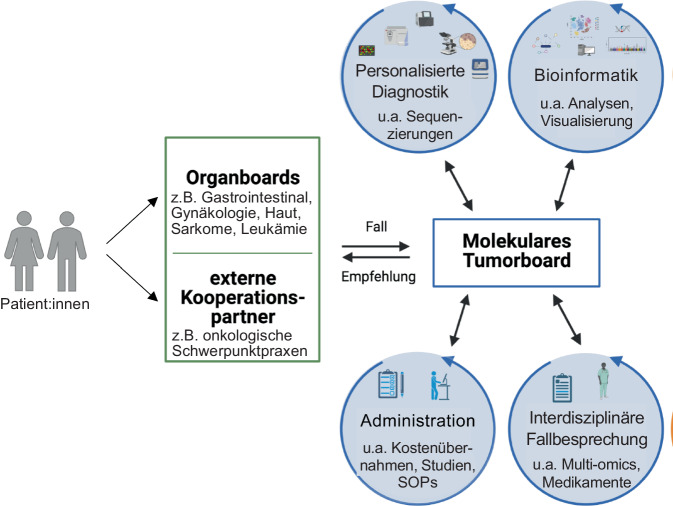
Standardisierter Arbeitsprozess in Molekularen Tumorboards (MTBs). *SOPs* Standard Operating Procedures. Quelle: eigene Abbildung

Hauptziel von MTBs ist es, Daten über spezifische molekulare Mechanismen von Krebserkrankungen in Kombination von klinischen Informationen zu nutzen und evidenzbasierte Therapieempfehlungen zu erarbeiten. Durch die Analyse individueller Patient:innendaten können Muster und Zusammenhänge identifiziert werden, die zur Entwicklung wirksamerer personalisierter Therapieansätze beitragen [2, 5, 9]. Für MTBs wurde ein standardisierter Arbeitsprozess, erarbeitet durch die Deutsche Krebsgesellschaft in Zusammenarbeit mit den Zentren für Personalisiert Medizin (ZPM), etabliert, der in vielen Krebszentren in ähnlicher Weise umgesetzt wird (Abb. 1).

Patient:innen werden in der Regel zunächst im Rahmen von organspezifischen Tumorkonferenzen (hier konzentriert sich die Expertise auf Tumorarten eines bestimmten Organs, z. B. Lunge, Gehirn, Prostata, oder einer Organgruppe, z. B. Gastrointestinaltrakt oder gynäkologische Tumore) oder über externe Kooperationspartner (z. B. niedergelassene Onkolog:innen) an das MTB überwiesen. Dort wird in der Regel eine personalisierte Diagnostik mit anschließender bioinformatischer Analyse, die erneut im MTB vorgestellt wird, angesetzt. Die unterschiedlichen Daten, sogenannte Multi-Omics-Daten, werden im MTB zur Unterstützung der Entscheidungsfindung präsentiert, was schließlich zu einer gemeinsamen Therapieempfehlung führt. Zur Unterstützung und Gewährleistung eines standardisierten Ablaufs und der Umsetzung der Empfehlungen benötigt jedes MTB auch eine administrative Einheit, die nicht nur die Kommunikation zu den externen Kooperationspartnern aufrechterhält, sondern auch die Verhandlungen zur Kostenübernahme mit den Krankenkassen und notwendige organisatorische Abläufe (SOPs) des MTB unterstützt bzw. übernimmt.

Die Empfehlungsrate für personalisierte Therapien der MTBs variiert je nach Art der molekularen Diagnostik und Verfügbarkeit von klinischen Studien bzw. Off-Label-Therapien und liegt zwischen 30 % und 50 %. In einigen MTBs liegt sie sogar bei über 80 %. Die stetig wachsende Zahl zugelassener Biomarker-stratifizierter Therapien trägt ebenfalls zur zunehmenden therapeutischen Relevanz einer umfassenden molekularen Diagnostik bei [10]. So konnten die Wirksamkeit und der klinische Nutzen einer molekulardiagnostisch gesteuerten personalisierten Therapie, die von einem MTB empfohlen wurde, sowohl in prospektiven als auch in retrospektiven Studien nachgewiesen werden [11–17].

## Herausforderungen für die Personalisierte Medizin und die Rolle von PM4Onco

Eine große Herausforderung für die PM und die Molekularen Tumorboards sind die Bereitstellung, Zusammenführung und Auswertung komplexer klinischer und molekulargenetischer Daten. Um genetische, molekulare und klinische Daten effizient erheben und nutzen zu können, ist die Einführung einheitlicher Datenstandards von entscheidender Bedeutung. Dies betrifft Formate für genomische Sequenzdaten, klinische Patient:innendaten, Therapieinformationen und vieles mehr [18]. Einheitliche Standards erleichtern nicht nur die Speicherung und Übertragung von Daten, sondern ermöglichen auch einen reibungslosen Austausch zwischen verschiedenen Institutionen und Systemen.

Das Verbundprojekt Personalisierte Medizin für die Onkologie (PM4Onco) wurde gegründet, um diese Herausforderungen anzugehen und die Zusammenarbeit zwischen bestehenden Initiativen und Netzwerken zu fördern. Ziel von PM4Onco ist es, eine Infrastruktur zu schaffen, die es ermöglicht, Daten aus der klinischen Versorgung und der Grundlagenforschung effizient zu nutzen, um die personalisierte Onkologie zu verbessern und voranzutreiben. PM4Onco umfasst nicht nur alle 4 Konsortien der Medizininformatik-Initiative (MII)^1^, DIFUTURE (Data Integration for Future Medicine), HiGHmed, MIRACUM (Medizininformatik in Forschung und Versorgung in der Universitätsmedizin) und SMITH (Smart Medical Information Technology for Healthcare), sondern baut auch auf bereits etablierten Strukturen, wie z. B. dem MII-Kerndatensatz, bestehend aus Basis- und Erweiterungsmodulen,^2^ auf und entwickelt diese weiter. Der MII-Kerndatensatz enthält wesentliche Elemente zur strukturierten und standardisierten Erfassung von Gesundheitsdaten und ist eine zentrale Voraussetzung für die gemeinsame Nutzung von Daten im Gesundheitswesen. PM4Onco unterstützt zum einen die Implementierung und Weiterentwicklung der bestehenden Erweiterungsmodule Onkologie, Pathologie-Befund und Molekulargenetischer Befundbericht und erarbeitet zum anderen das Erweiterungsmodul Molekulares Tumorboard, basierend auf den klinischen und genetischen Kerndatensätzen der ZPM/DNPM. Diese Erweiterungsmodule in Kombination bilden das Anwendungsprofil „Personalisierte Onkologie (PersOnco)“. Auf diese Weise wird PM4Onco zur Schaffung einer vernetzten und datengesteuerten Gesundheitslandschaft und zu einer personalisierten und effizienten Gesundheitsversorgung beitragen und gleichzeitig den medizinischen Fortschritt und die Forschung fördern.

Ein weiterer wichtiger Aspekt für die PM sind die individuellen Krankheitsverläufe einzelner Patient:innen. Hierbei spielt die Nutzung von Krebsregisterdaten eine entscheidende Rolle. Diese ermöglichen in der Onkologie einen umfassenden Einblick in den Verlauf der Krebserkrankung der einzelnen Patient:innen. Die Verfolgung des Krankheitsverlaufs über einen längeren Zeitraum ermöglicht ein detailliertes Follow-up der Patient:innen während oder nach abgeschlossener Therapie und ist besonders wichtig, um Rückfälle zu erkennen, Langzeitwirkungen zu überwachen und die Langzeitüberlebensraten zu analysieren. Dies fördert nicht nur die Forschung und Entwicklung neuer Therapieansätze, sondern trägt auch zur kontinuierlichen Verbesserung der onkologischen Versorgung bei. Aus diesem Grund arbeitet PM4Onco mit der Plattform § 65c, einem Expertengremium, das einen dauerhaften fachlichen Austausch der klinischen Krebsregister nach § 65c SGB V gewährleistet, zusammen, um die Daten der klinischen Krebsregister zu nutzen und zu integrieren.^3^

Zur Erfassung weiterer individuelle Daten von Patient:innen außerhalb des Krankenhausaufenthaltes wird PM4Onco das patientenzentrierte Befragungsinstrument der „Patient Reported Outcome Measures“ (PROMs) nutzen. Auf diese Weise können z. B. Aussagen zur Lebensqualität, zum Auftreten von Symptomen oder zur Zufriedenheit direkt durch die Patient:innen erfasst werden [19]. Dies ist in der Onkologie besonders wichtig, da Krebstherapien häufig mit Nebenwirkungen verbunden sind. Im Kontext der Personalisierten Onkologie spielen PROMs eine entscheidende Rolle und tragen zu einem ganzheitlichen Verständnis der Patient:innenerfahrung bei. Ebenso können durch die regelmäßige Erfassung von PROMs Probleme und Einschränkungen frühzeitig erkannt und somit rechtzeitig geeignete Maßnahmen ergriffen werden, um die Lebensqualität der Patient:innen zu verbessern und Nebenwirkungen der Behandlung zu minimieren. PROMs dienen daher als wichtige Parameter zur Bewertung von Therapieerfolgen aus Sicht der Patient:innen. Verbesserungen oder Verschlechterungen der Lebensqualität und des Wohlbefindens können als zusätzliche Endpunkte in klinischen Studien und Therapieevaluationen berücksichtigt werden. Aus diesem Grund wird sich PM4Onco für die digitale Umsetzung und Erfassung dieser Daten in die Datenintegrationszentren (DIZ) widmen, um sie effektiv für Versorgung und Forschung zugänglich zu machen.

## Infrastruktur und Konsortien

Verschiedene onkologische Konsortien und Initiativen mit unterschiedlichen Schwerpunkten haben sich mittlerweile gebildet, um die onkologische Versorgung und Forschung voranzutreiben. PM4Onco führt diese Initiativen und Konsortien zusammen, um gemeinsam „unter einem Dach“ Standards sowie die Bereitstellung und Auswertung von Daten zu optimieren mit dem Ziel, personalisierte Therapieansätze präziser und effizienter zu gestalten. Im Rahmen der Medizininformatik-Initiative (MII) wurden in der Aufbau- und Vernetzungsphase (2018–2023) bereits der klinische Use Case „Onkologie“ mit dem HiGHmed-Konsortium^4^ und der infrastrukturell und methodische Anwendungsfall „From Knowledge to Action – Unterstützung für das Molekulare Tumorboard“ mit dem MIRACUM-Konsortium^5^ umgesetzt. Die Erfahrungen und geschaffenen Strukturen der MII, wie z. B. die Datenintegrationszentren, ermöglichen in der Ausbau- und Erweiterungsphase (2023–2026) die Etablierung des Verbundprojektes PM4Onco, das durch die „Nationale Dekade gegen Krebs“^6^ unterstützt und finanziert wird.

**Abb. 2:**
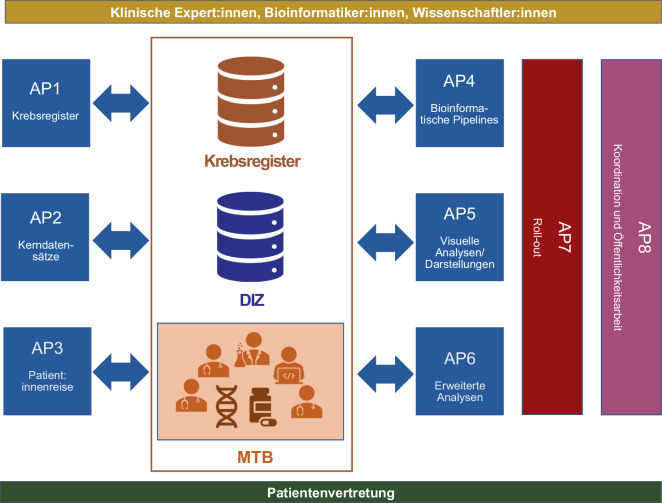
Struktur der Arbeitspakete (AP) im Verbundprojekt PM4Onco. Quelle: eigene Abbildung. Abkürzungen: *DIZen* Datenintegrationszentren, *MTB* Molekulares Tumorboard

PM4Onco umfasst 23 Standorte und fast 200 Mitglieder. Es schließt alle onkologischen Spitzenzentren ein und profitiert dabei von den bereits erprobten klinischen Strukturen und Prozessen des Bayerischen Zentrums für Krebsforschung (BZKF)^7^, des Deutschen Netzwerks Personalisierte Medizin (DNPM; [20]), des „DKFZ/NCT/DKTK MASTER Program“^8^ [2, 5], des nationalen Netzwerks Genomische Medizin (nNGM; [21]), der Zentren für Personalisierte Medizin (ZPM; [22]) sowie der Nationalen Strategie für Genommedizin (genomDE)^9^. M. Boerries (Koordinatorin) sowie B. Brors und O. Kohlbacher (Co-Koordinatoren) koordinieren das Verbundprojekt, das aus 8 Arbeitspaketen (AP1–AP8) besteht, die jeweils von einer Leitung und einer Co-Leitung betreut werden (Abb. 2). Sie arbeiten gemeinsam an den Aufgaben, koordinieren den Fortschritt in den einzelnen Arbeitspaketen (AP) und treiben das Projekt voran.

*AP1 (Integration mit Daten aus Krebsregistern)* konzentriert sich auf die wichtige Datenmodalität der nationalen Krebsregister. Hier wird durch die Beteiligung der Plattform § 65 c nicht nur die Abstimmung der verschiedenen Krebsregister harmonisiert, sondern es können zugleich auch Prozesse und Standards über die Plattform national bereitgestellt und verteilt werden. In diesem AP werden auch die Erfahrungen der Tumordokumentar:innen genutzt, um Umsetzungsmöglichkeiten in der Routine der Dokumentation bestmöglich anzuwenden. Ein Fokus wird auch auf der Bereitstellung von Plausibilitätsregeln für eine bessere Datenqualität (insbesondere in Bezug auf molekulargenetische Daten für Tumorboards) liegen.

*AP2 (Standardisierte Kerndatensätze)* widmet sich der Standardisierung und Harmonisierung klinischer und molekulargenetischer Daten aus der personalisierten Onkologie. Es wird ein Erweiterungsmodul „Molekulares Tumorboard“ (MTB) erstellt, das mit dem nationalen Kerndatensatz des MII kompatibel ist und die standardisierten Datendarstellungen bereits bestehender personalisierter Krebsnetzwerke wie DKFZ/NCT/DKTK MASTER, ZPM und DNPM berücksichtigt. Hierbei wird eine Absprache und Zusammenarbeit mit der Taskforce Kerndatensatz der MII-Arbeitsgruppe Interoperabilität^10^ stattfinden.

Das übergeordnete Ziel von *AP3 (Patient:innenreise)* ist die sektorenübergreifende Optimierung des Patient:innenpfades/der Patient:innenreise unter Einbeziehung von Kliniker:innen, Ärzt:innen und Patient:innen durch den verstärkten Einsatz der Digitalisierung. Insbesondere sollen Interaktionen wie Patient:innenbeteiligung und -information gestärkt werden, um die Eigenverantwortung der Patient:innen zu fördern. Hierzu werden unter anderem Fragebögen (auch in Bezug auf Lebensqualität) definiert, um spezifische klinische Prozesse abzudecken, die für das MTB relevant sind.

**Abb. 3:**
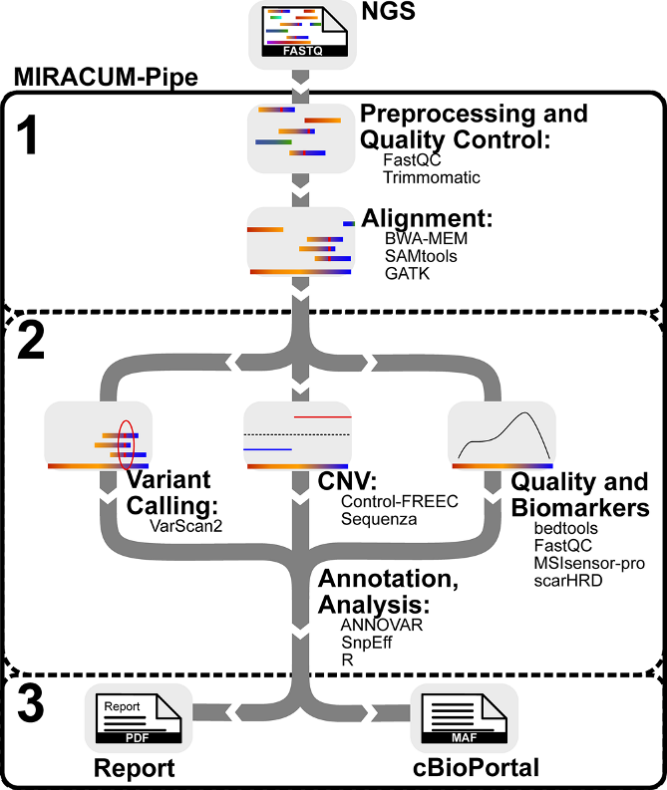
Schematische Darstellung der MIRACUM-Pipe. MIRACUM-Pipe gliedert sich in 3 Hauptteile: (*1*) Vorverarbeitung, Qualitätskontrolle und Alignment, (*2*) Analyse, Annotation und Interpretation, unterteilt in Variantenaufruf (VC), Kopienzahlvariationen (CNV) sowie Qualitätsbewertung und Biomarkerberechnung, und (*3*) Zusammenführung der Ergebnisse. (Anmerkungen: *NGS* Next-Generation Sequencing; *cBioPortal* Visualisierungsplattform für u. a. genomische Daten vom „Memorial Sloan Kettering Cancer Center“ in New York, USA. Die sonstigen Begriffe, wie z. B. FastQC, bwa-mem, sind Eigennamen von verwendeten Programmen). Quelle: eigene Abbildung, publiziert [23]

Dafür wird insbesondere die Erstellung eines Integrationskonzeptes für die patient:innenbezogenen Eingangsdaten in die DIZ wichtig sein. *AP4 (Standardisierte Bioinformatik- und Dateninfrastrukturen)* sammelt, vergleicht und standardisiert Bioinformatik-Pipelines, die für molekulare Tumorboards verwendet werden, und evaluiert und definiert die entsprechende Dateninfrastruktur [23–25]. Ein Beispiel ist die MIRACUM-Pipe, die eine einfach zu bedienende, standardisierte Lösung zur Analyse von Sequenzierungsdaten, einschließlich Qualitätskontrolle, Varianten-Calling, Kopienzahlveränderungen, Annotation, Visualisierung und Berichterstellung, anbietet (Abb. 3; [23]).

Besonderes Augenmerk liegt auf Pipelines, die eine umfassende genomische Charakterisierung ermöglichen, wie zum Beispiel Ganzgenom- und Gesamtexomsequenzierung. Hierbei werden bioinformatische Ringversuche mit Sequenzierungsdaten durchgeführt [24], die bekannte Varianten enthalten. Zudem erfolgt die Bestimmung geeigneter Metriken, um Pipelines miteinander zu vergleichen. Das übergeordnete Ziel besteht darin, die Transparenz von Entscheidungsprozessen, die auf der Bestimmung von Varianten basieren, zu verbessern. Dies wird durch die Durchführung einer einheitlichen statistischen Bewertung der Variantenbestimmung erreicht, unter vollständiger Berücksichtigung aller damit verbundenen Unsicherheiten.

*AP5 (Visuelle Analysen/Darstellungen)* fokussiert sich auf die interaktive Visualisierung komplexer Patient:innendaten. Die rasanten Fortschritte in der Sequenzierung und Analyse führen zu einer rapiden Zunahme der Möglichkeiten und Datenmengen in der personalisierten Onkologie. Eine entscheidende Rolle spielt dabei die Visualisierung dieser Daten, um komplexe molekulare Informationen rasch zu verstehen und zu vergleichen und damit optimale Diagnosen und Therapieentscheidungen zu ermöglichen [26, 27]. Dies erfordert eine agile Entwicklung und wiederholte Neubewertungen aller klinischen und computergestützten Akteure, um scheinbar neue Daten bestmöglich zu verarbeiten, zu integrieren und darzustellen. Die visuelle Analyse soll krankenhausübergreifend standardisiert sein und sich auf Basis einer FHIR-basierten Integration an neue Technologien und Daten anpassen lassen. Die Darstellung, Suche und der Vergleich von Patient:innendaten müssen auf verschiedenen Endgeräten und in der klinischen Praxis intuitiv bedienbar sein. AP5 baut diesbezüglich auf bestehenden Data Warehouses für personalisierte Onkologie und Krebsstudien wie cBioPortal oder AMBAR auf, die die Basis für Plugin-basierte Erweiterungen entsprechend den technologischen und klinischen Anforderungen bilden [28–32]. Die Benutzerfreundlichkeit und Anforderungen dieser Visualisierungstools werden anhand von Anforderungsanalysen bewertet [27, 28].

In *AP6 (Erweiterte Analyse)* werden fortgeschrittene Analyseverfahren, die über die bioinformatische Standardverarbeitung klinischer und genomischer Daten (AP4) hinausgehen und für die zukünftige Einbindung in die MTB-Diskussionen relevant sein könnten, validiert und qualitätskontrolliert implementiert. Darüber hinaus wird die Anwendung von Methoden der künstlichen Intelligenz nicht nur die Analyseverfahren verbessern, sondern auch die Identifizierung von neuen Biomarkern und komplexen Biomarkersignaturen oder von Patienten mit ähnlichen Krankheitsbildern und Therapieverläufen („Patient-like-me“) ermöglichen, um Tumore genauer zu charakterisieren und gleichzeitig neue Therapieoptionen zu eröffnen.

*AP7 (Roll-out)* hat das Ziel, die notwendigen technischen Komponenten und organisatorischen Maßnahmen von PM4Onco in den beteiligten DIZ zu implementieren. Da Letztere aus allen 4 Konsortien der MII stammen, weisen sie (leicht) unterschiedliche technische Architekturen auf. Um diesem Aspekt Rechnung zu tragen, wird ein 2‑stufiger Implementierungsprozess stattfinden, bei dem zunächst 2 führende DIZ pro MII-Konsortium die notwendigen konsortialspezifischen ETL-Prozesse und -Komponenten sowie die notwendige Dokumentation für den Betrieb und die Wartung der Softwarelösungen entwickeln (Phase-I-DIZ). Alle Standorte der Phase-I-DIZ verfügen zugleich über ein etabliertes MTB und können somit einen großen Erfahrungsschatz an Prozessen, Strukturen und Infrastrukturen einbringen. Diese DIZ werden die notwendigen Komponenten und Prozesse für eine reibungslose schrittweise Einführung in den Phase-II-DIZ vorbereiten und unterstützen.

Das *AP8 (Kommunikation und Öffentlichkeitsarbeit)* beinhaltet das Projektmanagement und stellt somit sicher, dass die Meilensteine, Ziele und Leistungen erbracht und erreicht bzw. die vertraglichen Verpflichtungen des Verbundprojektes erfüllt werden. Das Arbeitspaket ist für die Kommunikation sowohl innerhalb des Projekts als auch nach außen (Öffentlichkeitsarbeit) zuständig und ist dabei angehalten, die Aspekte von Gender und Gleichberechtigung explizit zu berücksichtigen. Ein wichtiger Teil von AP8 ist zudem die Einbeziehung der Patient:innenvertretung: Über den Bundesverband *Haus der Krebs-Selbsthilfe e.* *V.* konnten 2 Patientenvertreter gewonnen werden, die das Projekt kontinuierlich begleiten und die Sicht der Betroffenen aktiv einbringen werden. Beide Vertreter sind ständige Gäste des Steuerungsgremiums und haben somit die Möglichkeit zur umfassenden Information und die Gelegenheit, sich in jedes Arbeitspaket und Themengebiet aktiv einzubringen.

Nicht zuletzt soll im Themengebiet „Schulung“ der wissenschaftliche Nachwuchs frühzeitig eingebunden werden, um die Interaktion und Vernetzung zu fördern. Für dieses Vorhaben wurden 4 Nachwuchsforschungsgruppen der MII ausgewählt. Die Sprecher:innen dieser Gruppen sind zum einen als ständige Gäste zu den Sitzungen des Steuerungsgremiums eingeladen, um die Aufgaben zur Koordination und Lenkung eines Verbundprojekts direkt mitzuerleben. Zum anderen organisieren die 4 Nachwuchsforschungsgruppen jeweils eigenständig einen Workshop, der thematisch sowohl an ihren eigenen Forschungsschwerpunkt als auch an eines der Arbeitspakete von PM4Onco anknüpft. Die Nachwuchswissenschaftler:innen übernehmen dabei die gesamte Organisation, von der Akquise der Sprecher:innen bis zur praktischen Durchführung vor Ort. Diese praktische Vernetzungsarbeit zielt darauf ab, langfristig grundlegende und wichtige Strukturen für die Personalisierte Medizin aktiv aufzubauen und zu stärken.

## Fazit

PM4Onco strebt eine Allianz zwischen der MII, den onkologischen Spitzenzentren und Konsortien, den klinischen Krebsregistern, Nachwuchswissenschaftler:innen, Patient:innen und Bürger:innen an, um die Personalisierte Medizin in der Krebstherapie zu stärken. Durch die Integration von Spitzentechnologie, umfassender Datenanalyse und interdisziplinärer Zusammenarbeit werden Fortschritte erzielt, die dazu beitragen, die Herausforderungen der Krebsbehandlung auf individueller Ebene zu bewältigen.

## Footnotes

1 https://www.medizininformatik-initiative.de/de/konsortien. Zugegriffen: 22.04.2024.

2 https://www.medizininformatik-initiative.de/de/der-kerndatensatz-der-medizininformatik-initiative. Zugegriffen: 22.04.2024.

3 https://plattform65c.de. Zugegriffen: 22.04.2024.

4 https://www.highmed.org/de/highmed-use-case-onkologie. Zugegriffen: 22.04.2024.

5 https://www.miracum.org/forschung/archiv/use-cases/use-case-3-from-knowledge-to-action. Zugegriffen: 22.04.2024.

6 https://www.dekade-gegen-krebs.de. Zugegriffen: 22.04.2024.

7 https://bzkf.de. Zugegriffen: 22.04.2024.

8 DKFZ/NCT/DKTK MASTER (Molecularly Aided Stratification for Tumor Eradication Research) Program: Programm des Deutschen Krebsforschungszentrums (DKFZ), des Nationalen Centrums für Tumorerkrankungen Heidelberg (NCT) und des Deutschen Konsortiums für Translationale Krebsforschung (DKTK).

9 https://www.bundesgesundheitsministerium.de/themen/gesundheitswesen/personalisierte-medizin/genomde-de. Zugegriffen: 22.04.2024.

10 https://www.medizininformatik-initiative.de/de/zusammenarbeit/arbeitsgruppe-interoperabilitaet. Zugegriffen: 22.04.2024.

## References

[1] Nolan E, Lindeman GJ, Visvader JE (2023). Deciphering breast cancer: from biology to the clinic. Cell.

[2] Mock A, Teleanu MV, Kreutzfeldt S (2023). NCT/DKFZ MASTER handbook of interpreting whole-genome, transcriptome, and methylome data for precision oncology. NPJ Precis Oncol.

[3] Möhrmann L, Werner M, Oleś M (2022). Comprehensive genomic and epigenomic analysis in cancer of unknown primary guides molecularly-informed therapies despite heterogeneity. Nat Commun.

[4] Hoefflin R, Geißler AL, Fritsch R (2018). Personalized clinical decision making through implementation of a molecular tumor board: a German single-center experience. JCO Precis Oncol.

[5] Horak P, Klink B, Heining C (2017). Precision oncology based on omics data: the NCT Heidelberg experience. Int J Cancer.

[6] Tamborero D, Dienstmann R, Rachid MH (2022). The molecular tumor board portal supports clinical decisions and automated reporting for precision oncology. Nat Cancer.

[7] Hoefflin R, Lazarou A, Hess ME (2021). Transitioning the molecular tumor board from proof of concept to clinical routine: a German single-center analysis. Cancers.

[8] van der Velden DL, van Herpen CML, van Laarhoven HWM (2017). Molecular tumor boards: current practice and future needs. Ann Oncol.

[9] Stenzinger A, Moltzen EK, Winkler E (2023). Implementation of precision medicine in healthcare—a European perspective. J Intern Med.

[10] Suehnholz SP, Nissan MH, Zhang H (2018). Quantifying the expanding landscape of clinical actionability for patients with cancer. Cancer Discov.

[11] O’Dwyer PJ, Gray RJ, Flaherty KT (2023). The NCI-MATCH trial: lessons for precision oncology. Nat Med.

[12] Bayle A, Belcaid L, Aldea M (2023). Clinical utility of circulating tumor DNA sequencing with a large panel: a national center for precision medicine (PRISM) study. Ann Oncol.

[13] Trédan O, Wang Q, Pissaloux D (2019). Molecular screening program to select molecular-based recommended therapies for metastatic cancer patients: analysis from the ProfiLER trial. Ann Oncol.

[14] Conley BA, Doroshow JH (2014). Molecular analysis for therapy choice: NCI MATCH. Semin Oncol.

[15] Rodon J, Soria JC, Berger R (2019). Genomic and transcriptomic profiling expands precision cancer medicine: the WINTHER trial. Nat Med.

[16] Massard C, Michiels S, Ferté C (2017). High-throughput genomics and clinical outcome in hard-to-treat advanced cancers: results of the MOSCATO 01 trial. Cancer Discov.

[17] Sicklick JK, Kato S, Okamura R (2021). Molecular profiling of advanced malignancies guides first-line N-of-1 treatments in the I-PREDICT treatment-naïve study. Genome Med.

[18] Prokosch HU, Acker T, Bernarding J (2018). MIRACUM: medical informatics in research and care in university medicine. Methods Inf Med.

[19] Gräßel L, Kruszewski M, Kuehn J (2022). Quality of life and patient satisfaction of participants of the molecular tumour board Freiburg—a single-center cross-sectional analysis.

[20] Illert AL, Stenzinger A, Bitzer M (2023). The German network for personalized medicine to enhance patient care and translational research. Nat Med.

[21] Büttner R, Wolf J, Kron A (2019). Das nationale Netzwerk Genomische Medizin (nNGM): Modell für eine innovative Diagnostik und Therapie von Lungenkrebs im Spannungsfeld eines öffentlichen Versorgungsauftrages. Pathologe.

[22] Stenzinger A, Edsjö A, Ploeger C (2022). Trailblazing precision medicine in europe: a joint view by genomic medicine Sweden and the centers for personalized medicine, ZPM, in Germany. Semin Cancer Biol.

[23] Metzger P, Hess ME, Blaumeiser A (2023). MIRACUM-pipe: an adaptable pipeline for next-generation sequencing analysis, reporting, and visualization for clinical decision making. Cancers.

[24] Menzel M, Ossowski S, Kral S (2023). Multicentric pilot study to standardize clinical whole exome sequencing (WES) for cancer patients. NPJ Precis Oncol.

[25] Bailey MH, Meyerson WU, Dursi LJ (2020). Retrospective evaluation of whole exome and genome mutation calls in 746 cancer samples. Nat Commun.

[26] Hinderer M, Boerries M, Haller F, Wagner S, Sollfrank S, Acker T, Prokosch HU, Christoph J (2017). Supporting molecular tumor boards in molecular-guided decision-making—the current status of five German university hospitals. Stud Health Technol Inform.

[27] Buechner P, Hinderer M, Unberath P, Metzger P, Boeker M, Acker T, Haller F, Mack E, Nowak D, Paret C, Schanze D, von Bubnoff N, Wagner S, Busch H, Boerries M, Christoph J (2020). Requirements analysis and specification for a molecular tumor board platform based on cbioportal. Diagnostics.

[28] Renner C, Reimer N, Christoph J (2023). Extending cBioPortal for therapy recommendation documentation in molecular tumor boards: development and usability study. JMIR Med Inform.

[29] Cerami E, Gao J, Dogrusoz U (2012). The cBio cancer genomics portal: an open platform for exploring multidimensional cancer genomics data. Cancer Discov.

[30] Gao J, Aksoy BA, Dogrusoz U (2013). Integrative analysis of complex cancer genomics and clinical profiles using the cBioPortal. Sci Signal.

[31] Fürstberger A, Ikonomi N, Kestler AMR (2023). AMBAR—interactive alteration annotations for molecular tumor boards. Comput Methods Programs Biomed.

[32] Unberath P, Mahlmeister L, Reimer N, Busch H, Boerries M, Christoph J (2022). Searching of clinical trials made easier in cBioPortal using patients’ genetic and clinical profiles. Appl Clin Inform.

